# The application of a new clear removable appliance with an occlusal splint in early anterior crossbite

**DOI:** 10.1186/s12903-021-01393-7

**Published:** 2021-01-21

**Authors:** Jiayu Zhang, Yuzhi Yang, Xue Han, Tingting Lan, Fei Bi, Xiangchen Qiao, Weihua Guo

**Affiliations:** 1 State Key Laboratory of Oral Diseases, West China Hospital of Stomatology, Sichuan University, No. 14, 3rd Sec. Ren Min Nan Road, Chengdu, 610041 Sichuan Province People’s Republic of China; 2 National Clinical Research Center for Oral Diseases, West China Hospital of Stomatology, Sichuan University, No. 14, 3rd Sec. Ren Min Nan Road, Chengdu, 610041 Sichuan Province People’s Republic of China; 3 National Engineering Laboratory for Oral Regenerative Medicine, West China Hospital of Stomatology, Sichuan University, No. 14, 3rd Sec. Ren Min Nan Road, Chengdu, 610041 Sichuan Province People’s Republic of China; 4 Department of Pediatric Dentistry, West China School of Stomatology, Sichuan University, No. 14, 3rd Sec. Ren Min Nan Road, Chengdu, 610041 Sichuan Province People’s Republic of China; 5 Chengdu Renjitiancheng Biotechnology Limited Corporation, Chengdu, People’s Republic of China

**Keywords:** Occlusal interference, Anterior crossbite, New clear removable appliance, Early treatment

## Abstract

**Background:**

The effectiveness of anterior crossbite treatment in preschool-aged children depends on the treatment design and patient compliance. Common early treatment appliances with steel wires and acrylic resin can bring about numerous problems, such as toothache, sore gums and mucous membrane injury. The aim of this study was to propose a new clear removable appliance to provide preschool-age children with an improved experience of early occlusal interference treatment.

**Methods:**

Appliances were designed with the help of 3-dimensional (3D) digital reconstruction oral models and fabricated using 3D printing technology and the pressed film method. Then, the mechanical properties of the original dental coping sheet and thermoformed aligners were assessed in a simulated intraoral environment. Preschool-age participants who displayed anterior crossbite were recruited in this study. Records (photographs and impressions) were taken before the treatment (T1), during the treatment (T2) and at the end of the treatment (T3). The effects of treatment were evaluated by clinical examination and questionnaires.

**Results:**

Normal degrees of overbite and overjet in the primary dentition were achieved using this new appliance. Dental and soft tissue relationships were improved. Questionnaires showed that the safety evaluation, degree of comfort and convenience grades of the appliance were all relatively high.

**Conclusion:**

This explorative study demonstrates that our new clear removable appliance is able to correct early-stage anterior crossbite in a safe, comfortable, convenient and efficient way. Thus, it is a promising method to correct a certain type of malocclusion, and its clinical use should be promoted in the future.

## Background

Malocclusion has become a major oral health issue of interest among preschool-age children [1, 2]. According to epidemiological surveys conducted by different countries, malocclusion among preschool children exhibited substantial variability, with the prevalence ranging from 45 to 80% [2–4]. In mainland China, 45.50% of children aged 2–7 years had at least one malocclusion [5].

Once occlusal interference occurs, soft tissue damage such as gingival trauma follows [2, 6]. In addition, the position of the mandibular condyle changes immediately after the onset of occlusal interference [7]. Without proper and timely orthodontic intervention, the condyle will gradually adapt to a new position, which may be pathological. On the one hand, soft tissues, especially muscle, undergo functional reconstruction on account of adaptation. This is likely to cause changes in craniomaxillofacial morphology and airway condition, among other features. As a result, the nervous system and muscles generate feedback that gives rise to the formation of malocclusion [8]. On the other hand, secondary growth of bone tissue caused by stretching or squeezing of soft tissue will lead to the abnormal development of both the maxilla and mandible, aggravating the deformity [9]. In the long term, occlusal interference brings about various types of malocclusion, and it may even affect the balance of the temporomandibular joint and cause temporomandibular disorders. Malocclusions in primary dentition are an urgent problem that is attracting increasing attention [10].

Among all types of occlusal interference, anterior crossbite (including an edge-to-edge relationship) has an especially profound effect and needs to be corrected immediately [11, 12]. The mandible displays considerable growth potential, presenting a higher and later growth peak than the maxilla. Therefore, if the crossbite hinders the growth of the maxilla, incompatibility between the maxilla and mandible will appear and become exacerbated over time. Abnormal structure and function of the lateral pterygoid are mostly correlated with Class III malocclusion [13], which may hint that early crossbite is likely to cause temporomandibular disorders.

**Fig. 1:**
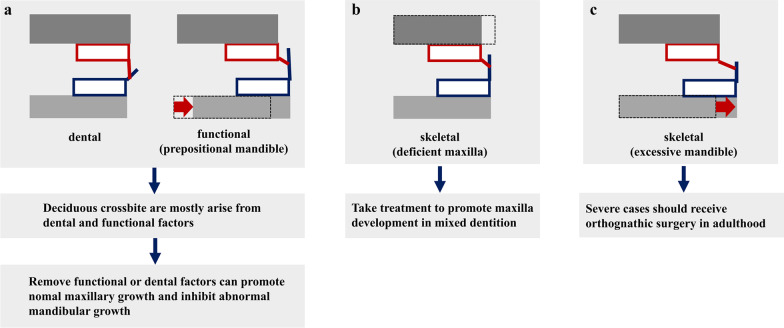
Orthodontic strategy for Class III malocclusion. **a** Treatment strategies for dental and functional Class III malocclusion. **b** Treatment strategies for skeletal Class III malocclusion with a deficient maxilla. **c** Treatment strategies for skeletal Class III malocclusion with an oversized mandible

It is possible to eliminate crossbite at an early age and minimize the future risk of severe malocclusions by providing appropriate treatment in a timely manner [14, 15]. Utilizing growth potential to promote normal maxilla and mandible development can help achieve an ideal profile, avoiding complex orthodontic treatment and orthognathic surgery in the future [16, 17] (Fig. 1).

Many studies have reported on the challenges of crossbite therapy and Class III therapy. Considering the age of the children and the extent to which they are likely to co-operate, the common form of treatment uses removable appliances with lingual springs or labial bow [14, 15]. These appliances consist of three major parts that play different functional roles: Adams cribs or hooks, which work as retentive components to provide stability; posterior occlusal splints, which serve as a tool to open the bite; and lingual springs, which act as an augmenter to relieve crossbite. However, the pungent smell of acrylic and the fatigue breakage of steel wires make this approach uncomfortable and dangerous for preschool-age children, leading to poor compliance and low treatment efficiency [18, 19]. In addition, frequent follow-up visits are required to trim the posterior bite splint and enhance the retention force.

The use of clear aligners in orthodontic treatment makes it possible to produce an appliance without steel wires or acrylic resin. Given that the specific, concrete pattern of an individual’s growth and development is difficult to predict precisely, orthodontic practices used to apply this technology mostly to permanent dentition, which is relatively stable [20]. However, when the aim is simple malocclusion correction with little growth change during the treatment, the range of aligners can be expanded to deciduous dentition and mixed dentition, making them appropriate for early occlusal interference correction. However, existing clear aligners cannot be directly applied. Making certain appropriate adjustments is essential for our treatment strategy.

In this article, we propose a new clear aligner therapy and meticulously illustrate its mechanical properties, describe the therapeutic process, and evaluate its effect on two qualified patients. The aim of this study is to propose a new removable appliance without steel wires or acrylic resin to solve the problems mentioned above, providing preschool-age children with a better experience of early occlusal interference treatment.

## Methods

### Characteristics of the new appliance

As thermoplastic polymers are highly viscoelastic materials, temperature, humidity, time after elastic deformation, and forming procedures have marked effects on their mechanical properties [21]. However, material manufacturers provide only the physical values of the standard sample measured under standard atmospheric conditions. There has never been a report on the physical values of these thermoformed aligners detected in simulated intraoral environments. The mechanical properties of the original dental coping sheet (Scheu-Dental, Iserlohn, Germany) and thermoformed aligners in a simulated intraoral environment were tested; the groups are listed in Table 1.

**Table 1:** Materials used in this study

Grouping	Treatment
Original dental coping sheet	Prior to thermoforming, the standard specimens were conditioned at room temperature (23 °C) for 24 h
Thermoformed aligners under standard atmospheric conditions	After thermoforming, the specimens for clinical use were conditioned at room temperature (23 °C) for 24 h
Thermoformed aligners in a simulated intraoral environment	After thermoforming, the specimens for clinical use were immersed in normal saline at 37 °C for 24 h

Tensile tests were performed with a multifunctional statics experimental machine (Instron) at room temperature. At least three specimens were tested in each of the three groups mentioned above. In order to obtain stress–strain curves, the distance between set-points was defined as 20 mm, and the crosshead speed was defined as 5 mm/min. The elastic modulus and tensile yield stress were calculated from the resulting stress–strain curves.

For the stress-relaxation measurements, specimens were stretched to 20% strain at a strain speed of 5 mm/min at room temperature. The maximal stress relaxation was termed the initial stress (N0), while the stress relaxation measured at t = 60 min was set as the residual stress (N1). Statistical significance among groups was tested by one-way ANOVA.

### Participants

Preschool-age participants who displayed anterior crossbite were recruited from West China Hospital of Stomatology.

The treatment protocol was approved by the ethics committee of West China Hospital of Stomatology and accepted by parents who chose this new appliance for their children. Parents gave written informed consent to participate in the study. As the study is a case series analytical study, no sample size calculation was undertaken. The inclusion criteria were as follows:

- Participants with clinically diagnosed anterior crossbite in their primary dentition
- Participants with the ability to co-operate and receive treatment from clinicians

### Design of a new appliance

Existing clear aligners are mostly applied in the correction of malocclusion in permanent dentition. Therefore, they cannot be directly applied without modification. Making certain appropriate adjustments is essential for our treatment strategy. It is a core principle that the bite should be opened to make room for the relief of occlusal interference, which cannot be achieved with current clear aligners. Thus, we designed digital prototypes with bumps on the posterior teeth to accomplish our first improvement of the device. The occlusal surface and the height of the bite splint both depend on the individual case. All parts of the appliance are included in one single-layer plastic film wrapping the dentition.

**Fig. 2:**
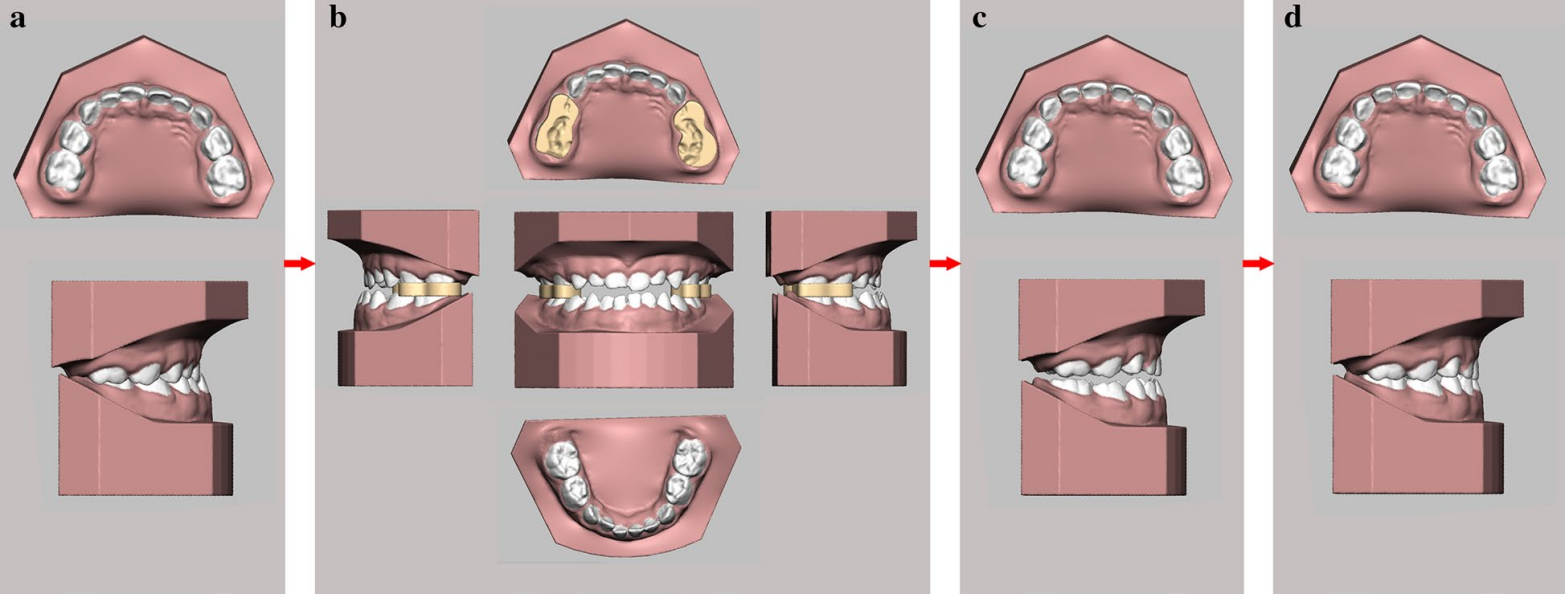
Simulating the tooth movement process with the digitally reconstructed oral model. **a** The initial digital model. **b** Building bite splints on the digital model. **c** Simulating tooth movements in the digital model using 3D animation software. **d** The final digital model

**Fig. 3:**
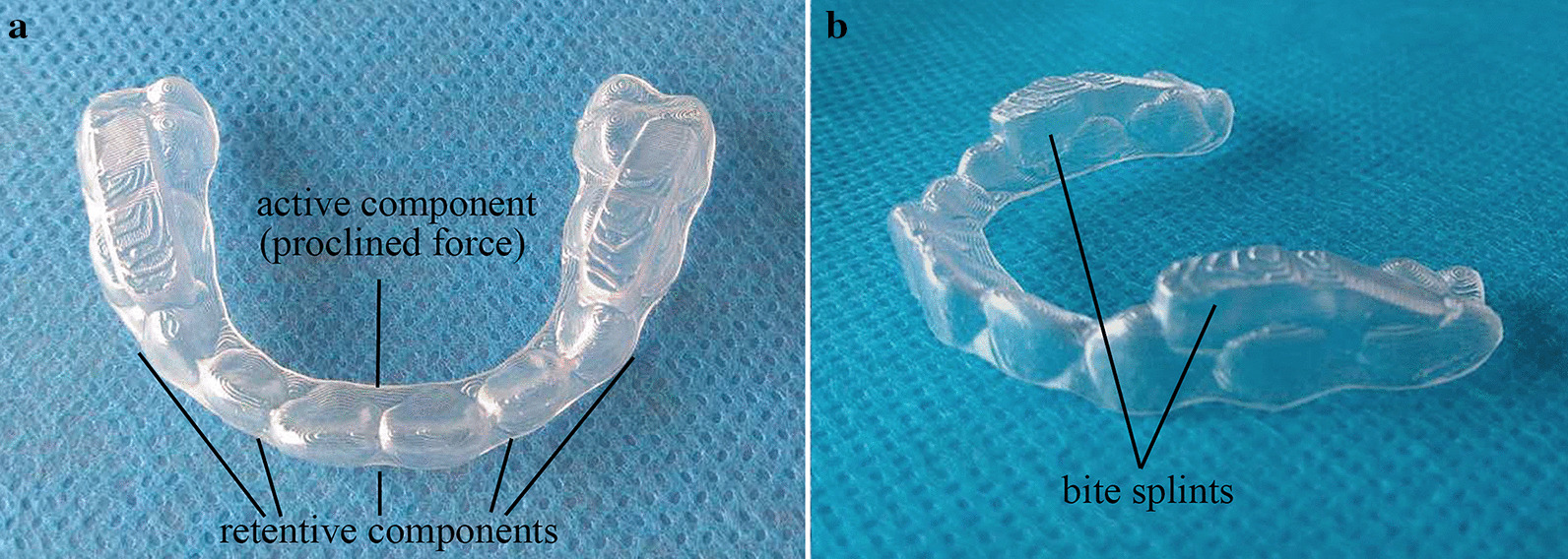
The composition of a new clear removable appliance without steel wire. **a** The structure of the appliance. **b** Bite splints on the posterior teeth

For anterior crossbite patients, the aligners are designed to procline the upper anterior teeth and retrude the mandible. In most cases, clear aligners can easily achieve the goal of tilting the target teeth. The whole therapeutic process was animated by the software Romexis 3D Ortho Studio (Planmeca, Finland) in a 3D model for clear visualization of tooth movement (Fig. 2). After certain modifications were made, this design was exported as an STL file and sent to a print service to obtain a series of casts with posterior occlusal splints through 3D printing technology (iSLA660, ZRapid Tech, China). Then, aligners were fabricated on a sequence of casts by the pressed film method (Fig. 3). The unique construction of this new aligner focused on building occlusal splints in the posterior area to open the bite and applying force to the anterior part to relieve the crossbite. Through a change in the vertical dimension of occlusion, the lower incisors could arc back more in line with the upper anterior teeth [22]. The rotation of the mandible can improve the profile. It also separates the lower teeth from the interfering upper labial inclines that force the mandible forward [22]. In addition, the condylar growth response is related to the function of the lateral pterygoid muscle [23, 24]. The frequency of pathological changes of the lateral pterygoid muscle (LPM) is especially high in patients with Class III malocclusion [13]. The elimination of interference permits the muscles to relax from their state of spasm [22].

To retard material abrasion originating from chewing, we added steps, reduced the amount of movement between each step and asked the patients to change the aligner every week. In the near future, we will try to develop new materials and apply 3D printing technology to the manufacture of aligners with solid bite splints.

### Treatment process

First, dentition impressions and photos were taken by clinicians to record participants’ facial and dental condition (T1). The new clear aligners were designed and fabricated on the basis of impressions; therefore, a precise impression was the first step towards success. In view of the characteristics of children, the use of alginate impression material instead of silicon rubber can both speed the process of making impressions and greatly reduce discomfort for the patient. Second, we made a cast from the impression as quickly as possible, avoiding deformation of the alginate impression material. The cast was scanned and digitized by a laser surface scanning system (iTero Element; Align Technology). Then, the STL files were imported into Romexis 3D Ortho Studio (Planmeca, Finland) and reconstructed automatically into a digital oral model in software. If conditions allowed, these two steps could be combined with intraoral scanning. Third, a series of steps, such as dental model analyses, alignment of digital dental models, and digital treatment planning, were carried out in the software. Next, a cast with bumps on the posterior teeth was obtained to produce thermoformed aligners by the film-pressing method. Finally, the clinician gave the appliance to the patients’ parents and showed them how to apply, remove, and clean the device. The patients were asked to wear appliances all day, except when brushing their teeth after each meal. Patients' condition was recorded during (T2) and after the treatment (T3).

### Clinical examination

For each patient, the effect of this new aligner therapy was assessed after treatment. The criteria of normal overbite and overjet in primary dentition were defined as overbite of 0–5 mm and overjet of 0–4 mm [25]. The total treatment time was recorded. Considering the age of the patients, radiographic examination and analysis were not performed to avoid excess radiation.

### Questionnaires

The safety, comfort and convenience of the appliance were graded using questionnaires (Additional file 1), which were completed by the patients and their parents. Each item was graded from one point (very poor) to ten points (very good).

## Results

### Mechanical properties

**Fig. 4:**
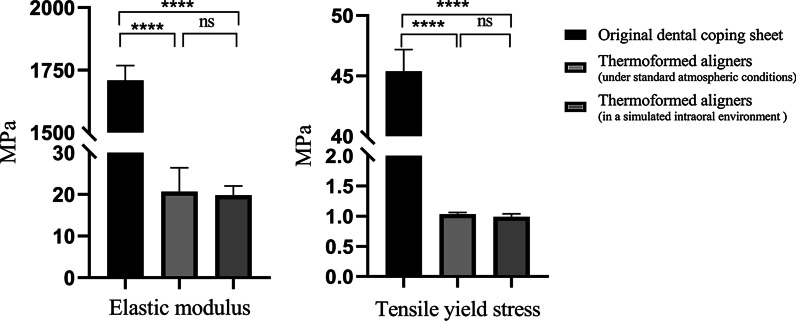
Comparison of elastic moduli and tensile yield stress in three sets of samples

**Fig. 5:**
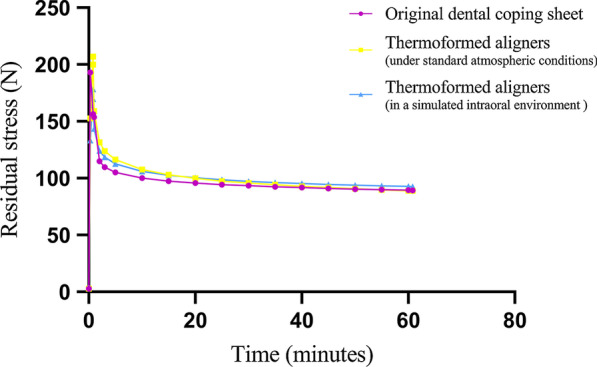
Stress relaxation of three sets of samples

The elastic modulus and tensile yield stress are shown in Fig. 4. Thermoformed specimens showed significant reductions in their elastic modulus and tensile yield stress compared to untreated dental coping sheets. However, no obvious distinctions were observed between the two treated groups. For the stress-relaxation measurements, the curves of the three sets of samples showed the same tendency (Fig. 5). The stress generated by a given strain decayed over time due to the viscoelasticity of thermoplastic polymers. The ranking of initial stress (N0), from highest to lowest, was as follows: original dental coping sheet, thermoformed aligners under standard atmospheric conditions, and thermoformed aligners in a simulated intraoral environment. The residual stress (N1) of all sets after an hour was maintained at approximately 100 N.

### Case presentation

We report the effects of a new clear aligner therapy in two very young patients with anterior crossbite. The details are as follows:

### Case I

**Fig. 6:**
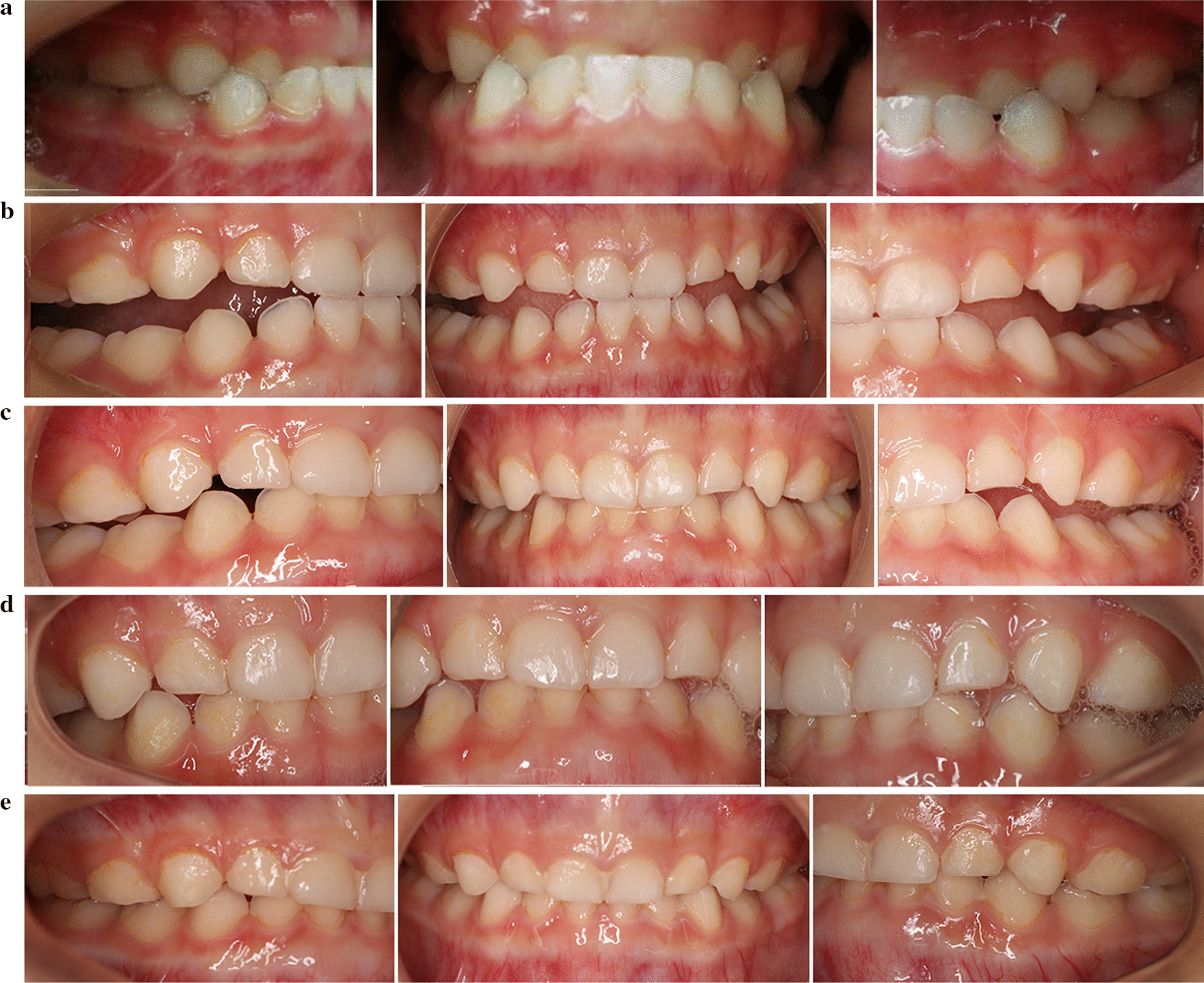
The comparisons of Case I records. **a** Pretreatment intraoral photographs (T0). **b** After 2 weeks of treatment (T1-A). **c** After 8 weeks of treatment (T1-B). **d** After 14 weeks of treatment (T1-C). **e** Posttreatment intraoral photographs (T2)

A 4-year-old female patient was brought in for anterior crossbite treatment. Facial photographs revealed a concave facial profile, a protrusive chin, everted lips, and a normal facial midline. Intraoral examinations revealed mesial terminal plane relationships on both sides and a deep crossbite in the anterior segment (Fig. 6a). A functional mandibular protrusion habit was detected, but there were no symptoms of temporomandibular disorders. Furthermore, her father showed similar extraoral and intraoral conditions, indicating a family history. Based on these findings, the patient was diagnosed with Class III malocclusion, deep anterior crossbite, and occlusal interference.

### Case II

**Fig. 7:**
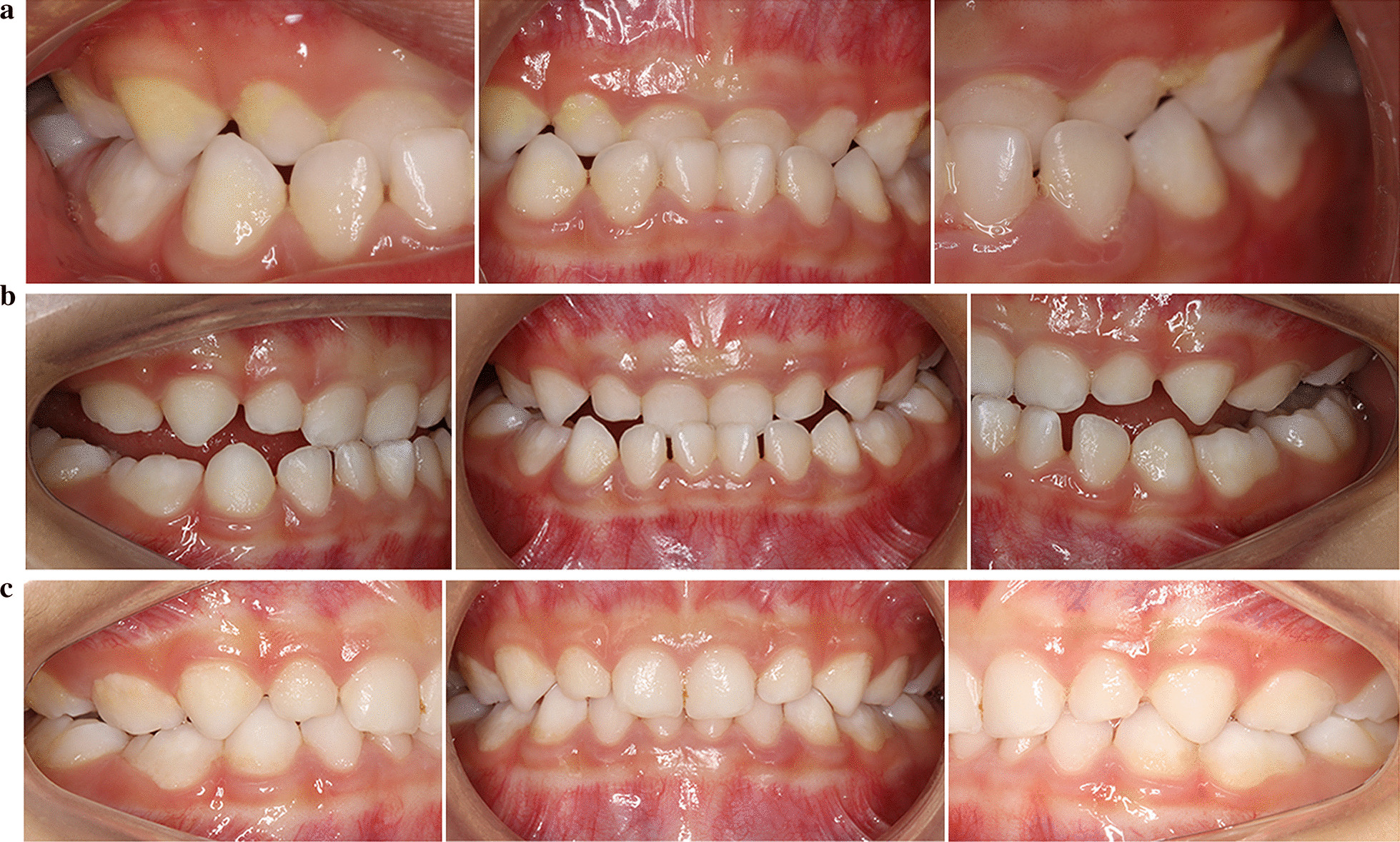
The comparisons of Case II records. **a** Pretreatment intraoral photographs (T0). **b** After 14 weeks of treatment (T1). **c** Posttreatment intraoral photographs (T2)

A 3-year-old male patient was brought in for anterior crossbite treatment. Facial photographs revealed a straight profile and a normal facial midline. Intraoral, dental cast, and 3D model examinations showed mesial terminal plane relationships on both sides and a moderate crossbite in the anterior segment (Fig. 7a). No symptoms of temporomandibular disorder were detected. His parents stated that the child had an incorrect nursing habit of lying flat while drinking from a bottle and had a functional mandibular protrusion habit. In this case, there was no evidence of a family history of Class III malocclusion. Based on these findings, the patient was diagnosed with a Class III malocclusion, moderate anterior crossbite, and occlusal interference.

Based on the data described in the two clinical cases, the first treatment option was a conventional appliance with steel wire. However, both patients were unwilling to wear the appliance because it was uncomfortable and even painful. In response, the new clear removable appliance was chosen. We designed and fabricated new clear aligners following the treatment protocol mentioned above and started treatment as early as possible. After several hours of adaptation, patients were able to speak and eat with the appliance. According to their parents' statement, the new appliance was easier to wear than a conventional appliance and comfortable for the child during the period of home treatment. After 2–6 months of treatment, the crossbite was basically corrected. The patients’ upper anterior teeth were proclined, and the anterior crossbite was corrected. Then, the subsequent aligners were designed with thinner bite splints, allowing the posterior teeth to erupt gradually. The overall treatment time for the primary dentition was 4–8 months.

After clinical examination, normal overbite and overjet in the primary dentition were achieved using this new appliance. Proper condylar position was achieved by eliminating occlusal interference and repositioning of the mandible, preventing future temporomandibular disorders. Based on a comparison between T3 records and T1 records, dental and soft tissue relationships were improved (Figs. 6, 7). Questionnaire results (Table 2) showed that during the therapeutic process, the treatment was safe and comfortable for the children and saved time for the parents. After the appliance was removed, regular dental visits were suggested for long-term observation of oral health.

**Table 2:** Satisfaction with the new appliance

Items	Grade (0–10)
Feeling of security	10
Feeling of comfort	9.5
Convenience	10
Early adaptability of appliance	8.5
Correction time	9
Pronunciation while wearing appliance	10
Appearance of appliance	10

## Discussion

**Fig. 8:**
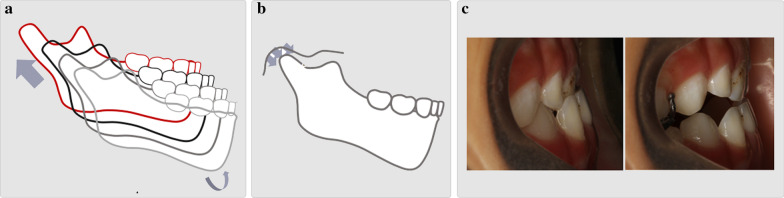
Growth and development characteristics of condyles. **a** The natural growth tendency of the mandible (the secondary growth of the condyle). **b** The secondary growth of the condyle can be restrained by applying compressive force. **c** Clinical treatment using conventional appliances has already shown that bite opening is beneficial for improving the profile

**Fig. 9:**
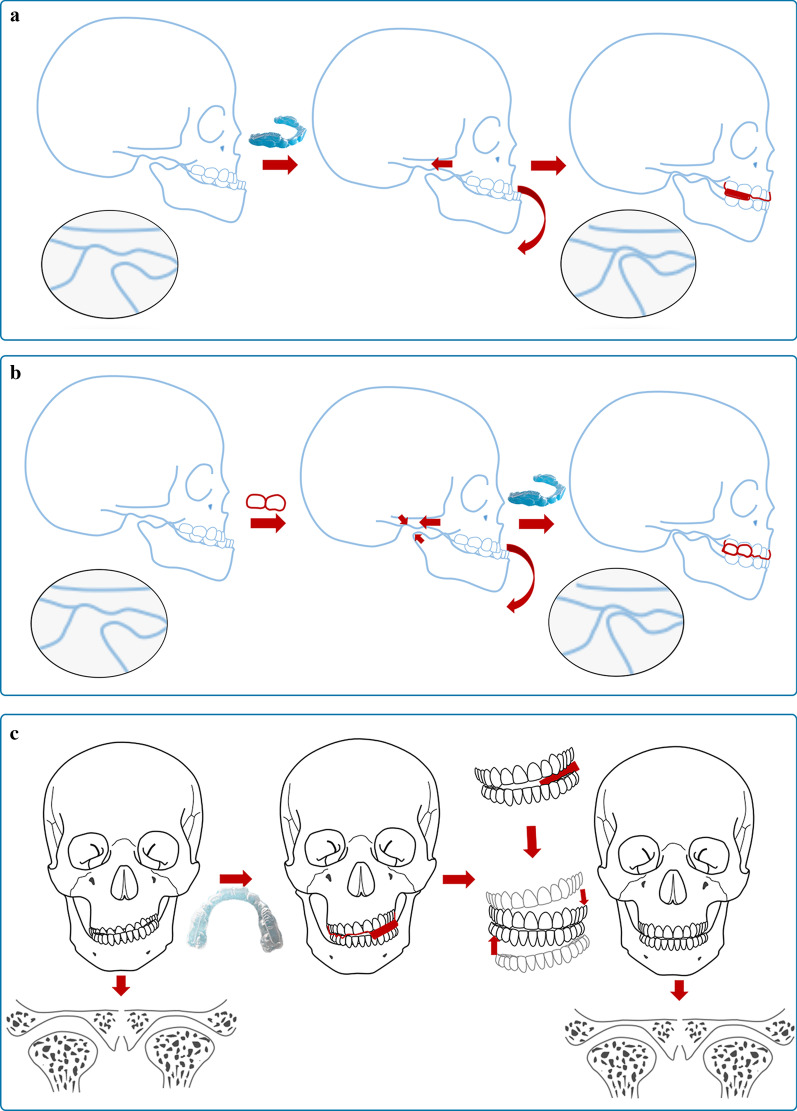
The application of occlusal surfaces and the height of the bite splint in different cases. **a** The application of aligners without anatomical structures on the occlusal surface. **b** The application of aligners with anatomical structures on the occlusal surface. **c** The application of a nonuniform bite splint

Effective treatment influences the way occlusal interference develops and reduces the complexity of subsequent treatment. The right choice of appliance for this treatment period is therefore of utmost importance [11, 12, 26]. At present, most clinical treatments in adults are symptomatic and rely on dental compensation rather than skeletal development. In deciduous dentition, mandibular ossification depends on endochondral and periosteal activity. The secondary growth of the condyle can be restrained by applying compressive force [27]. Based on the mechanical and biological characteristics of mandibular growth, repositioning the mandible can inhibit the growth of the mandible by adjusting the three-dimensional direction of the jaw and occlusion [28]. Additionally, the correct position can help adjust the functional status of the upper and lower lateral pterygoid muscles [29]. In this way, not only dental problems but also skeletal deformities can be corrected or alleviated. Clinical treatment using conventional appliances has already shown that bite opening is beneficial for improving the profile (Fig. 8). In the new clear aligner therapy, a posterior bite splint is included in one single-layer plastic film wrapping the dentition. It is decided on a case-by-case basis whether the occlusal surface capping the posterior bite has anatomical structures. In most cases with only occlusal interferences and no skeletal deformity, once the bite is opened, the mandible will assume the natural position by muscle stretching; in such cases, we can use aligners without anatomical structures on the occlusal surface. For children with slight skeletal deformity, anatomical structures are used to apply additional force to the condyle to promote or inhibit mandibular growth. For patients with asymmetrically positioned condyles, the vertical position of the condyle at the articular fossa can be adjusted by a bite splint of nonuniform height (Fig. 9).

In addition to appliance design, the mechanical properties of the new clear removable appliance were explored. In the results of these tests, the mechanical properties of thermoformed specimens were significantly degraded compared to the properties before thermoforming. These differences may be due to the changes in the thickness and shape of the original sheets after thermoforming, as there is a strong positive correlation between the mechanical properties and the thickness of sheets. The mechanical properties of thermoplastic materials can also be influenced by structural factors, environmental factors and heat history [21]. However, no significant changes were observed in thermoformed specimens under different temperature and humidity conditions, which illustrated that the intraoral environment had little influence on the orthodontic forces of those thermoformed aligners. The residual stress reminded us that the appliance could provide continuous light force in the intraoral environment.

In our case report, patients achieved satisfying dental and soft tissue relationships. The suitable height and rigidity of the bite splints prevented occlusal interference. The opening of the bite helped eliminate muscle memory to allow the patient's condyle to adjust to a posterior position, which should be much healthier, by keeping the mandible positioned accordingly [30]. Without the restriction imposed by the mandible, the favourable development of the maxilla was accelerated, which was beneficial for skeletal changes [31].

The clear aligners relieved the dental phobia of young children due to the small size and comfortable material. These devices helped the patients receive orthodontic treatment in a timely manner, promoting the normal development of the maxilla and mandibula to reduce treatment difficulty in the future. Timely treatment corrects improper condylar positioning and prevents temporomandibular disorders. Furthermore, the aligners can be sequentially exchanged by the patients at home. With no need for frequent adjustment at the office, both the practitioners and the families of the patients save time. The simple treatment protocol makes it possible to promote early correction of occlusal interference in remote areas. Clinicians can collect data and impressions from children troubled by occlusal interference during epidemiological surveys, then design and manufacture all the aligners and send the appliances to those remote areas by express. It is believed that this system will soon become a reality when mature technology and supporting facilities come into being. The success of this new appliance is of great value in providing early dental care for children, benefiting all young children who need timely, comfortable, and reliable correction of occlusal interference.

However, there are still some deficiencies in our study. First, additional studies must be carried out to confirm the effectiveness of the new appliance for not only anterior crossbite but also other types of occlusal interference. Second, according to the principles of design, precision is important in every detail. At present, aligners are mostly made by the pressed film method, which is less accurate than 3D printing. The next step is to develop new materials and applying 3D printing technology to the fabrication of aligners as soon as possible. In this way, it will be possible to manufacture precisely shaped aligners with solid bite splints.

## Conclusion

This new clear removable appliance with an occlusal splint is worth promoting vigorously for early treatment occlusal interference. The simple protocol and comfortable treatment experience can relieve children's fear and enhance treatment efficiency by improving patient co-operation. Not only anterior crossbite but also other regular forms of occlusal interference, such as posterior crossbite, overbite and overjet caused by a narrow maxillary arch, can also be corrected in this way. Removing the interference in a timely manner ensures appropriate condylar position, which contributes to joint health and normal skeletal development. Overall, the application of clear aligners with bite splints provides children in urban and rural areas with equal opportunity to receive early interceptive orthodontic treatment, lowering the prevalence of malocclusion.

## Supplementary Information

**Additional file 1**. Satisfaction questionnaire with the new appliance.Satisfaction with the new appliance were graded using questionnaires, which were completed by the patients and their parents. Each item was graded from one point (very poor) to ten points (very good).

## Data Availability

The datasets used and/or analysed during the current study are available from the corresponding author on reasonable request.

## Abbreviations

3D: 3-Dimensional
T: Treatment
LPM: Lateral pterygoid muscle
