# Supplementary material for: The application of a new clear removable appliance with an occlusal splint in early anterior crossbite
Source: BMC Oral Health. 2021 Jan 21;21:36. doi: 10.1186/s12903-021-01393-7 (PMC7818763; doi:10.1186/s12903-021-01393-7)
Supplement: Supplementary file 1 — Additional file 1. Satisfaction questionnaire with the new appliance.Satisfaction with the new appliance were graded using questionnaires, which were completed by the patients and their parents. Each item was graded from one point (very poor) to ten points (very good). [file 12903_2021_1393_MOESM1_ESM.docx]

Supplementary file 1. Satisfaction questionnaire with the new appliance.

对矫治器满意度调查表

| Items  项目 | Grade (0-10) |
| --- | --- |
| Feeling of security  安全性 |  |
| Feeling of comfort  舒适性 |  |
| Convenience  方便性 |  |
| Early adaptability of appliance  患儿对矫治器早期适应性 |  |
| Correction time  对矫治时长满意度 |  |
| Pronunciation while wearing appliance  患儿佩戴矫治器时发音情况 |  |
| Appearance of appliance  对矫治器外观满意度 |  |
